# Factors Associated with Health Literacy among the Elderly People in Vietnam

**DOI:** 10.1155/2020/3490635

**Published:** 2020-03-25

**Authors:** Ho Van Hoa, Hoang Thi Giang, Pham Tuan Vu, Duong Van Tuyen, Pham Minh Khue

**Affiliations:** ^1^Faculty of Public Health, Haiphong University of Medicine and Pharmacy, Vietnam; ^2^Lao Tropical Medicine and Public Health Institute, Laos; ^3^Faculty of Nursing, Thai Nguyen University of Medicine and Pharmacy, Vietnam; ^4^School of Nutrition and Health Sciences, Taipei Medical University, Taipei, Taiwan

## Abstract

**Background:**

There is a lack of information regarding health literacy (HL) in elderly people in Vietnam.

**Objective:**

The aim of this study was to evaluate the health literacy and the associated factors in elderly people in Vietnam.

**Methods:**

A cross-sectional study was conducted on a sample of 300 elderly people aged 55 years and above. Data were obtained from study participants using face-to-face interviews using designed questionnaires on sociodemographics, behaviors, and health literacy. Multiple linear regression models were performed to identify potential determinants of health literacy.

**Results:**

HL scores were 29.70 ± 8.20 for the general HL dimension, 32.00 ± 9.60 for the healthcare dimension, 21.97 ± 10.06 for the disease prevention dimension, and 35.15 ± 9.43 for the health promotion dimension. In the final model, age was negatively associated with HL (*B* − coefficient = −0.09, 95% confidence interval (95% CI) (-0.17 to -0.008), *P* = 0.030). Occupation (*B* = 4.77, 95% CI (3.18 to 6.36), *P* < 0.001), taking care of children (*B* = 1.68, 95% CI (0.21 to 3.15), *P* = 0.025), social activity (*B* = 4.61, 95% CI (2.86 to 6.37), *P* < 0.001), doing exercises (*B* = 2.52, 95% CI (1.07 to 3.96), *P* = 0.001), television watching (*B* = 2.10, 95% CI (0.75 to 3.45), *P* = 0.002), using the Internet (*B* = 2.93, 95% CI (1.29 to 4.57), *P* = 0.001), and social connection (*B* = 3.50, 95% CI (1.23 to 5.78), *P* = 0.003) were positively associated with HL, respectively.

**Conclusion:**

Age, occupation, and a number of behaviors were significantly associated with HL in elder people. Health education campaigns should take into account the above factors as facilitating access to the Internet and providing opportunities for social networks for the elderly.

## 1. Introduction

Health literacy (HL) is a new concept in Vietnam as in many other Asian countries. It can be defined as “the degree to which individuals can obtain, process, and understand the basic health information and services they need to make appropriate health decisions” [1]. HL is a term introduced in the 1970s [2]; in addition to basic literacy skills (reading, writing vocabulary, spelling, and comprehension), HL requires knowledge on health topics. It refers to the capacity of people to answer their health needs in a modern society [3]. As a complex concept, it has had been defined in many different ways. This study uses a broad and inclusive definition proposed in 2012 by the European Health Literacy consortium (HLS-EU). This definition considers that HL is linked to literacy. It entails people's knowledge, motivation, and competencies to access, understand, appraise, and apply health information [4].

Low HL is a significant problem everywhere in the world, even in developed countries. For example, in the United States in 2003, approximately 80 million adults (36%) were estimated to have limited health literacy [5]. Rates of limited health literacy in certain population subgroups were higher [5]. This has been shown to be the case of the elderly, minorities, individuals who have not completed high school, adults who speak a language other than English before starting school, and people living in poverty [5]. In Europe, at least 1 out of 10 individuals (12%) was found in 2015 to have inadequate health literacy. However, the differences between states can be substantial: only 2% of Dutch people have an inadequate health literacy, compared to 27% in Bulgaria, for example [6]. The impact of HL is nontrivial. HL is associated with better health status, healthier behavior, and better accessibility and use of healthcare facilities [7].

### 1.1. Determinants of Health Literacy

The determinants of HL found in the literature are the level of education, which is positively correlated with HL [5], [8–11], income [5, 8, 11, 12], self-perceived social class belonging [8], and health-related behaviors such as involving in community activities, using the Internet, or watching television [13].

### 1.2. Health Literacy and the Elderly

Age has been found to be associated with HL level [14]. The ability of a person to understand information related to his own health can be affected by age-related physical and mental declines. Old people need some school-trained abilities to adequately use healthcare services, as the ability to read and calculate. The elderly has lower rates of Internet use compared with other adults [15]. For those reasons, older people are at a particularly vulnerable position regarding their health literacy capacity [14, 16].

One study has provided HL data for Vietnam. This study was performed in six Asian countries: Taiwan, Indonesia, Kazakhstan, Malaysia, Myanmar, and Vietnam. The general HL score of Vietnamese was found to be the lowest compared to the scores of the other countries [17]. Yet, this study did not discriminate old from younger people. Yet, the older subpopulation is an important group for which specific data is warranted. Elderly people have more health issues and are higher consumers of healthcare services, and their number is increasing. Indeed, the data of the 2006 National Wide Family Survey indicated that 95% of people who were 60 years old or more had health problems. On average, an elder has 2.69 diseases [18]. Moreover, in the survey, half of the elders self-reported that they had poor health status. This was particularly apparent in the elderly living in rural areas compared to those living in urban areas [19]. While several studies have demonstrated the prevalence of limited health literacy across the world [1, 20–23], data on health literacy among Vietnamese are still poor. Effectively, besides this study, HL topic has not been studied in Vietnam. There is therefore a lack of information regarding the importance of HL from a public health perspective.

Considering the low HL problems, its determinants among the elderly might differ from those found in younger age groups. Our study is aimed at exploring the HL level and its associated factors in Vietnamese older people.

## 2. Materials and Methods

### 2.1. Study Design

A cross-sectional study was conducted.

Data were collected between March and May 2018.

### 2.2. Study Location

We used both probability and nonprobability sampling techniques to draw the study samples. At first, we purposely chose the Yen Lac district because of its convenient accessibility. Yen Lac is located in the northern delta, on the left bank of the Red River (north bank of the river). It includes 16 communes with a total population of about 153 100 people. The majority of the population (90%) is concentrated in rural areas and works in agriculture (farming: paddy and maize; breeding: cattle, buffaloes, and pigs) [24]. The three following communes were chosen, on convenience grounds, for the data collection: Minh Tan, Nguyet Duc, and Van Tien. Minh Tan commune is located in the center of the district and is the district town; Van Tien and Nguyet Duc communes are two communes in the southwest and southeast of Minh Tan commune. The choice was aimed at allowing comparisons of HL between people living in urban and rural areas. In each commune, we randomly selected two villages.

### 2.3. Study Subjects

Data were collected on people aged 55 years or above. The elderly people are defined in Vietnam as people who are 60 years old or over. However in this study, we selected people who were 55 years old and above, as 55 is a threshold frequently used in HL studies [12, 14, 17, 25, 26]. To participate, people had to be willing to participate into this study and able to listen and understand Vietnamese.

### 2.4. Sample Size

G-power software version 3.1 [27] was used to calculate the sample size for multiple linear regression analyses, where the dependent variable is HL score. With expected *R*^2^ = 0.1, an alpha = 0.05, power = 0.95, and a number of potential predictors = 20, a minimum sample of 293 was deemed to be sufficient. We rounded the size to 300 individuals in order to have the same sample size in each of the three communes.

### 2.5. Data Collection Procedure

Due to time constraints, 3 interviewers participated in the data collection. Interviewers were trained before going to the field with a detailed protocol. Training was provided in order to reduce variability between all three of them.

The interviews took place at the respondent's house. The first older person was chosen randomly in the list of elderly people in the village. After the interview, the interviewer moved to the next house on the right side. If the elderly person was present in the household, he was solicited to participate to the study; if not, the interviews would continue moving to the next elderly. This way of constituting the sample continued until 100 participants per commune were obtained.

A consent form was given to the potential participants. After reading the document, the latter were able to discuss with the interviewer who answered any question they might have had. Interviews started once the consent form was signed. The questionnaire was read by an investigator who noted the answers provided by the participants in each question. An interview lasted about 30-40 minutes on average.

### 2.6. Research Instruments

Two questionnaires were used in this study: the health literacy short-form (HL-SF12) questionnaire and a sociodemographic and behavior questionnaire.

The sociodemographic questionnaire is aimed at collecting information on age (year of birth), gender (male and female), education level (no level completed, primary and lower secondary, and upper secondary), marital status (never married vs. married, divorced, and widowed), residence (urban or rural area), monthly income (≤1.3 million VND, >1.3-3 million VND, >3-5 million VND, and >5 million VND), and occupation (no occupation, worker, farmer, or house worker).

Behavioral variables were assessed, including doing housework (<1 time/week vs. ≥1 time/week), taking care of children or grandchildren (never, rarely, or <1 time/week vs. often or ≥1 time/week), doing unpaid voluntary work (<1 time/month vs. weekly to monthly), involving social activities (no vs. yes), doing sport or physical activities (less often or never vs. several days a week), attending courses, seminar, conferences or receiving private lessons (no vs. yes), listening to TV, radio, reading paper related to older people's health (rarely and almost every day), using information communication technology (ICT) (never vs. at least once a week), and social connection (rarely vs. almost every day).

Health literacy was measured using the HL-SF12 questionnaire [12].

This questionnaire contains 12 items: (1) find information on treatments of illnesses that concern you; (2) understand the leaflets that come with your medicine; (3) judge the advantages and disadvantages of different treatment options; (4) call an ambulance in an emergency; (5) find information on how to manage mental health problems like stress or depression; (6) understand why you need health screenings; (7) judge which vaccinations you may need; (8) decide how you can protect yourself from illness based on advice from family and friends; (9) find out about activities that are good for your mental well-being; (10) understand information in the media on how to get healthier; (11) judge which everyday behavior is related to your health; and (12) join a sports club or exercise class if you want to. The perceived difficulty of each health-related task was rated on 4-point Likert scales (1 very difficult, 2 difficult, 3 easy, and 4 very easy) [12]. HL-SF12 has been developed in Taiwan for assessing patients in general in 2015 [17] and six Asian countries for general public use [17]. It has not been used and validated in Vietnamese [12]. It allows calculating 4 scores: a general score and an individual score for the three dimensions of health: healthcare, disease prevention, and health promotion. Compared with the original instrument, it does not allow measuring four competencies, namely, the abilities to access, understand, appraise, and apply health information. HL-SF12 allows calculating the general HL index into unified metrics that range from 0 to 50 by using the formula index score = (*M* − 1)∗(50/3), where *M* is the mean of all 12 participating items for each individual (thus *M* ranging from 1 to 4), 1 is the minimal possible value of the mean, 3 is the range of the mean, and 50 is the chosen maximum value of the desired scale. 0 represents the lowest possible HL score and 50 as the highest possible score [28].

### 2.7. Data Analyses

Data were imported and analyzed using STATA v13.0 (Stata Corp, College Station, TX, USA). We used algorithms for descriptive statistics (percentage, mean, median, standard deviation, and range) to describe characteristics of participants. A simple linear regression model was then used to examine the association between the dependent variable (general HL score) and all the independent variables (age, sex, marital status, level of education, income, occupation, type of household, number of people in family, doing house work, taking care of children, social activities, physical exercise, attending the training lessons, listening TV/radio, using the Internet, and social connectedness). All variables with a significant level less than 20% were included into a multiple linear regression model to examine predictors of HL. The multiple regression proceeded through a stepwise backward approach in order to come up with a model as parsimonious as possible. The level of significance for keeping the variables was set at a *P* value of less than 0.05.

### 2.8. Ethical Consideration

This research proposal was approved by the Institutional Review Boards of the Hai Phong University of Medicine and Pharmacy, Vietnam (No. 15/HDDD).

## 3. Results

Table 1 presents the sociodemographic characteristics of the 300 participants. The mean age was 66.9 years ± 9.5 years with a range of 55-95 years. Only 4% were more than 85 years old. About half (54.00%) were women. Over two-thirds (69.3%) lived with a partner. More than half (60.3%) had a primary or lower secondary educational level. 47.3% had an income of less than 1.3 million VND per month. The mean number of people in the household was 5 ± 2. There were 27% of the participants who had not visited a doctor or a healthcare facility during the last 12 months.

Table 2 shows the social aspects of living that reflect individual activities, such as doing exercise, attending conferences, watching TV, using the Internet, and social connectedness. Our analysis showed that 84% of study participants were frequently doing their housework. Only 7.67% reported to have a voluntary unpaid job. The participating rate to a social activity (association of the elderly in the village) was as high as 82%.

Table 3 presents the scores of general HL and three dimensions: (1) healthcare HL, (2) disease prevention HL, and (3) health promotion HL. Vietnamese older people in Vinh Phuc district had the highest score of HL in health promotion (35.15 ± 9.43) while the lowest score on disease prevention.

Table 4 displays the findings from the multiple linear regression model on general HL and its potential determinants. Overall, the model shows that one year increase in age was associated with a reduction of -0.09 unit in HL score, with a *P* value of 0.03. In contrast, higher HL score was significantly observed in older people who had an occupation, took care of children, participated in social activities, did physical exercise frequently, listened to TV/radio, used the Internet, and had frequent social connection (*P* values < 0.01).

## 4. Discussion

In the literature, it has been shown that around the world, nearly 9 out of 10 adults may lack the skills needed to prevent the occurrence of preventable diseases and to contribute to an appropriate management of their health [5]. Effectively, adults with limited HL report less knowledge about their medical condition and treatment, worse health status, less understanding and use of preventive services, and a higher rate of hospitalization [22]. The problem of limited HL has been found to be the greatest for older adults [22].

Health literacy is a complex issue with numerous contributing factors. Importantly, these factors might be contextual. They might differ from one setting to another, hence the need to explore them in different places. This is necessary to identify the proper factors that, in each context, could become potential targets of public health programs aiming at improving HL [4].

Gen HL score found in our study was similar to the scores found in the previous research performed in Vietnam in 2014 [26]. The Gen-HL score that was found in this study (29.7 ± 8.2) is lower than that in other studies on the elderly. If this reflects a consequence of using a nonfully validated instrument in Vietnamese language or if this reflects real differences between Vietnamese and non-Vietnamese older people, this needs to be explored further on.

Healthcare HL and health promotion HL dimension scores were high while the disease prevention HL score was very low (21.97 ± 10.06). This result is important, as HL is a prerequisite to the effectiveness of prevention activities deployed by public health in the country. This underlines the relevance of exploring if public health interventions implemented in Vietnam are less effective in the older population. If yes, public health would have to revise its interventions in order to increase their effectiveness in this subgroup of the population.

When potential determinants of HL were analyzed, we found that age, occupation, taking care of children/grandchildren, social activities, doing sport, listening to TV/radio regarding older people's health, using ICT, and social connectedness might effectively impact the HL level.

The findings were similar to those found in previous studies that showed that HL is negatively associated with age. Older groups tend to have lower HL [14, 23, 26, 28]. This is an expected result because old people, particularly in developing countries, have a higher probability of having been deprived of schooling. Poor schooling negatively affects their literacy level. Yet, what our results show is that we should avoid considering all older people as a homogeneous group. Even among the elderly, there is a graduation. Public health interventions aiming at increasing HL among the old people should adapt to this heterogeneity. Specific intervention might be required for different groups of age even among the older people.

People who were still working tend to have a higher HL score. It is likely that working persons interact actively with other people, which whom they can share and discuss ideas, their feelings, and experiences. The colleagues can also provide new information. This has effectively been found in other studies [28, 29].

The proportion of the older people living with children or grandchildren was very high (81.67%) in the current study. Those living with descendants tend to have a higher HL, probably because discussion with people from a different generation increases the chance that someone gets new information or that his ideas are confronted. The intergenerational exchanges might potentially increase critical thinking, which is a factor that has been found to be associated with HL [30].

Participating in social activities was strongly positively associated with HL. Almost all people aged 55 and more participate to at least one activity for the elderly (82%) such as association of the elderly women union meetings (for women), sports game, and chess groups. Those are the places where the elderly can meet, discuss, and exchange their knowledge and experience with others. A positive association between participating in these activities and HL was therefore expected. Community involvement associated with higher health literacy was also found in Kazakhstan [31] and Taiwan [13].

Besides the fact that participating in social activities increased the possibilities to exchange information, one notes that some activities require some financial means. This is the case of many sports. We can raise the possibility that sports are also a confounding factor for the socioeconomic condition, which is known to be a determinant of HL [15, 32, 33].

Listening to the radio, watching TV, or reading newspapers, which are common in older people, were positively associated with HL. In Vietnam, as in many countries, the media are one of the principal sources of information used by public health for health education of the population. The positive association suggests that public health interventions implemented in the country are probably effective. One notes that this factor was also found positively significant in other research [7, 12, 13, 26, 31].

Using the Internet among elderly was low (26%), but those who used the Internet frequently have a higher HL. The Internet is a major tool for searching information. This result suggests that the Internet should be privileged in the future, considering the fact that the percentage of people familiar with its use is inversely correlated with age. As older people die, they are replaced by others who are a little younger and who are expected to be better users of the Internet [23, 34]. Using the Internet was found to have the positive effect on the care-seeking behavior in Vietnamese population [35].

Finally, in accordance with the above observations, social connectedness had a positive significant association with HL. Almost all the people in the sample had contact with others very regularly (91%). This high social life might be cultural. Vietnamese people like to be outside, to see neighbors, and to go to meetings. From a public health point of view, this indicates the importance of targeting communities, when trying to increase HL, and not only individuals.

## 5. Limitations

The most important limitation of our study is probably the fact that the HL questionnaire that was used had not been formally validated. We do not know if the concept of HL that we tended to measure is well captured by the wording of the questions. We cannot therefore exclude the possibility that a validated questionnaire would have brought different results. However, the fact that our results are coherent with the literature and the fact that the final regression model is statistically strong suggest that the translated questionnaires were a relevant source of information for such a study. Therefore, we can be confident in the results.

Moreover, we cannot exclude the fact that, in spite of the training provided, the use of 3 interviewers might have introduced some biases, as the specific interaction with a participant can influence his answer. Furthermore, it is possible that only the more enthusiastic and interested elderly people completed the survey. As stated above, the fact that sound results have been obtained suggests that the risk of such biases is very low. Finally, we must highlight the fact that a semiconvenience sample was constituted. We cannot ensure that it was representative of the population.

## 6. Implications

The elderly is a vulnerable group of the population. When people get older, their body declines. They more easily get diseases than young people do and they take more time to recover. With the increasing number of elderly year by year, the healthcare system cannot discard their importance. On the other hand, our results, from a public health perspective, are reassuring. The determinants found in this study are targets on which we can act. We know now that the Vietnamese old people like the media and that the media are an effective way to increase HL. Media should be used. We know now that the Internet is, speaking about HL improvement, an important factor to be considered. Public health must consider developing health education websites that are attractive to the old people who use the Internet. We know now that social interaction is an important and effective determinant of HL. Public health can increase its health education programs in places where old people meet and spend time. Health marketing can be focused on these places. In other words, our results suggest that public health can be more effective in increasing HL of the old people because there are targets that can be easily exploited.

## Figures and Tables

**Table 1:** Sociodemographic characteristics of participants (*N* = 300).

Variables	*n*	Percentage
Sex		
Male	138	46.0
Female	162	54.0
Living place		
Urban	100	33.3
Rural	200	66.7
Age in years		
55-64	146	48.7
65-74	82	27.3
75-84	60	20.0
≥85	12	4.0
Marital status		
Single	13	4.3
Married	208	69.3
Widowed	77	25.7
Divorced	2	0.7
Level of education		
No level completed	21	7.0
Primary and lower secondary	181	60.3
Upper secondary	98	32.7
Income		
≤1.3 million VND	142	47.3
>1.3–3 million VND	93	31.0
>3-5 million VND	54	18.0
>5 million VND	11	3.7
Occupation		
No occupation	92	30.7
Worker	92	30.7
Famer or house worker	116	38.7
Type of household		
Living alone or with someone about the same age	55	18.3
With children or/and grandchildren	245	81.7
Number of people in family		
Mean ± SD	4.9 ± 2.1	
≤2	58	19.3
3-5	104	34.7
≥6	138	46.0
Visiting doctors or healthcare facilities due to health problems		
Mean ± SD	2.7 ± 3.2	
Never	81	27.0
1-5 times	167	55.7
>5 times	52	17.3

**Table 2:** Frequency of activities that study subjects participated in (*N* = 300).

Variables	*n*	Percentage
Housework		
Less often (<1 time/week)	48	16.0
Often (≥1 time/week)	252	84.0
Taking care of children and grandchildren		
Less often (<1 time/week)	112	37.3
Often (≥1 time/week)	188	62.7
Doing unpaid voluntary work		
Almost never (<1 time/month)	277	92.3
Weekly or monthly	23	7.7
Participating to a social activity		
No	54	18.0
Yes	246	82.0
Doing sports or physical exercise		
Less often (<1 time/week)	164	54.7
Often (≥1 time/week)	136	45.3
Attending courses, seminars, and conferences or receiving private lessons		
No	252	84.0
Yes	48	16.0
Listening to TV and radio, reading paper regarding older people's health		
Less often (<1 time/week)	191	63.7
Often (≥1 time/week)	109	36.3
Using the Internet		
Never	222	74.0
At least once a week	78	26.0
Social connectedness		
Less often (<1 time/week)	27	9.0
Often (≥1 time/week)	273	91.0

**Table 3:** Health literacy scores.

Variables	Mean ± SD	Median	IQR
General HL	29.70 ± 8.20	29.20	24.30-36.10
Healthcare HL	32.00 ± 9.60	33.33	25.00-37.50
Disease prevention HL	21.97 ± 10.06	20.83	16.70-29.20
Health promotion HL	35.15 ± 9.43	33.33	29.20-41.70

**Table 4:** Multivariate linear regression analysis for health literacy (*N* = 300).

Variables	*B*-Coef	95% CI	*P* value
Age in years	-0.09	-0.17, -0.008	0.03
Occupation			
With no job^R^			
With job	4.77	3.18, 6.36	<0.001
Taking care of children, grandchildren			
Less often (<1 time/week)^R^			
Often (≥1 time/week)	1.68	0.21, 3.15	0.025
Social activities			
No^R^			
Yes	4.61	2.86, 6.37	<0.001
Doing sports or physical exercise			
Less often (<1 time/week)^R^			
Often (≥1 time/week)	2.52	1.07, 3.96	0.001
Listening to TV and radio, reading paper regarding older people's health			
Less often (<1 time/week)^R^			
Often (≥1 time/week)	2.10	0.75, 3.45	0.002
Using ICT			
Never^R^			
At least once a week	2.93	1.29, 4.57	0.001
Social connection			
Less often (<1 time/week)^R^			
Often (≥1 time/week)	3.50	1.23, 5.78	0.003

^R^Referent group. *F* (2, 125) = 31.53, Prob > *F* = 0.0001, *R*‐squared = 0.6248, Adj *R*‐squared = 0.6050, root MSE = 5.1553.

## Data Availability

The data used to support the findings of this study are available from the corresponding author upon request.
